# Thermo-Optical and Structural Studies of Iodine-Doped Polymer: Fullerene Blend Films, Used in Photovoltaic Structures

**DOI:** 10.3390/polym14050858

**Published:** 2022-02-22

**Authors:** Bożena Jarząbek, Paweł Nitschke, Marcin Godzierz, Marcin Palewicz, Tomasz Piasecki, Teodor Paweł Gotszalk

**Affiliations:** 1 Centre of Polymer and Carbon Materials, Polish Academy of Sciences, 34 M. Curie-Skłodowska Str., 41-819 Zabrze, Poland; mgodzierz@cmpw-pan.edu.pl; 2 Department of Nanometrology, Faculty of Electronics, Photonics and Microsystem, Wroclaw University of Science and Technology, 50-372 Wroclaw, Poland; marcin.palewicz@pwr.edu.pl (M.P.); tomasz.piasecki@pwr.edu.pl (T.P.); teodor.gotszalk@pwr.edu.pl (T.P.G.)

**Keywords:** polymer:fullerene blend films, iodine doping, annealing effect, absorption edge parameters, exciton bandwidth, structural changes, BHJ solar cells

## Abstract

Optical and structural properties of a blend thin film of (1:1 wt.) of poly(3-hexylthiophene) (P3HT) and [6,6]-phenyl-C61-butyric acid methyl ester (PCBM) doped with iodine (I_2_) and then exposed to a stepwise heating were reported and compared with the properties of doped P3HT films. The UV-Vis(*T*) absorption measurements were performed in situ during annealing runs, at the precisely defined temperatures, in a range of 20–210 °C. It was demonstrated that this new method allows one to observe the changes of absorption spectra, connected with the iodine release and other structural processes upon annealing. In addition, the thermally-induced changes of the exciton bandwidth (*W*) and the absorption edge parameters, i.e., the energy gap (*E*_G_) and the Urbach energy (*E*_U_) were discussed in the context of different length of conjugation and the structural disorder in polymers and blends films. During annealing, several stages were distinguished and related to the following processes as: the iodine escape and an increase in P3HT crystallinity, the orderly stacking of polymer chains, the thermally inducted structural defects and the phase separation caused by an aggregation of PCBM in the polymer matrix. Moreover, the detailed X-ray diffraction studies, performed for P3HT and P3HT:PCBM films, before and after doping and then after their thermal treatment, allowed us to consider the structural changes of polymer and blend films. The effect of iodine content and the annealing process on the bulk heterojunction (BHJ) solar cells parameters was checked, by the impedance spectroscopy (IS) measurements and the *J-V* characteristics registration. All of the investigated P3HT:PCBM blend films showed the photovoltaic effect; the increase in power conversion efficiency (PCE) upon iodine doping was demonstrated.

## 1. Introduction

Organic photovoltaic (OPV) systems have attracted much attention and have been intensively investigated in recent decades due to their advantages, such as: low production cost, light weight and mechanical flexibility [1]. Bulk-heterojunction (BHJ) PV cells, whose photoactive layers are composed of a blend of electron donating (donor-D) and electron accepting (acceptor-A) materials can maximize the interfacial D-A area, which allows for higher power conversion efficiencies of polymer OPV systems. A classic polymer donor, such as poly(3-hexylthiophene) (P3HT) blended with a fullerene derivative [6,6]-phenyl-C_61_-butyric acid methyl ester (PCBM) acting as an acceptor were widely investigated due to the broad absorption spectra and suitable energy gap, together with the good photo-generation of mobile charge carriers [2]. Nevertheless, the power conversion efficiency of this type of BHJ structure reaches only 5–5.5% [3,4] (while the state-of-the-art devices [5] currently reach over 18%) the different aspects of P3HT:PCBM blends are still presented in an enormous amount of publications. For many years, this type of polymer:fullerene structure has been recognized as a model system for the organic photovoltaic application, despite its rather low performance as an active layer [6]. In addition, other conjugated polymers based on polythiophenes (PTs) have been investigated in BHJ photovoltaic structures [7,8].

To improve the efficiency of these BHJ solar cells, many various post-depositions strategies were developed, such as: annealing (in the air or argon) films or substrates [4,9,10,11,12,13,14,15] and annealing solvents [2,15,16,17], with the controlled drying rate of obtained films [18,19]. Another way to improve photovoltaic properties (used both for non-organic semiconductors, as for conjugated polymers) is suitable doping. Among various dopants, halogens (Br, I, Cl) are one of the most often used doping agents. Iodine-doped-polymer films used in the light emitting diodes and/or solar cells were presented in [11,12,13], while for BHJ, structures this type of doping is also often described [20]. The iodine doping process may be realized from the gas phase, especially for the insoluble polymer films, obtained, e.g., by the CVD process [21,22], or in the case of soluble polymers, when thin films can be obtained using “wet” methods (spin-on, spray-on, printing); the doping process can be realized in solution, where the amount of doping factor can be precisely controlled [20].

In this work, we present the effect of stepwise, controlled annealing (up to 210 °C) of the iodine (I_2_)-doped (0, 1, 5, 10 mol.%) P3HT:PCBM (1:1) blend films, on the basis of in situ thermo-optical investigations. The same thermal treatment process was also used for the iodine-doped polymer (P3HT) films. This method of UV-Vis-NIR(*T*) measurements turned out to also be a very useful tool for investigations of the mesomorphic behavior of compounds [23] and to evaluate thermal stability of polymer thin films [24].

In our previous work [25], we investigated the behavior of neat P3HT and P3HT:PCBM blend films during annealing/cooling runs; several stages were distinguished and related to thermally inducted structural changes, using the similar thermo-optical studies.

Now, the main idea of this work was to check how the presence of iodine, as a doping factor, changes the properties of both pure polymer and blended polymer with fullerene thin films, also at the higher temperatures. It was demonstrated that the presence of PCBM affected the polymers behavior, both after doping and during annealing. These changes were discussed on the basis of absorption edge parameters (*E*_G_, *E*_U_) and the exciton bandwidth (*W*), obtained as a function of temperature, for a different % of the iodine content. Moreover, the detailed X-ray diffraction studies of polymer and blends films, before and after doping and annealing, allowed us to describe the changes of structural order and to confirm our explanation of thermo-optical results. To check the influence of doping and thermal treatment on BHJ solar cells with the P3HT:PCBM active layer, the impedance spectroscopy (IS) measurements and the current density-voltage (*J-V*) characteristics, together with solar cells parameters, were presented.

## 2. Materials and Methods

### 2.1. Materials

Poly(3-hexylthiophene) (P3HT, M102, M_n_ = 66 225 g/mol), [6,6]-Phenyl-C61-butyric acid methyl ester (PCBM, M111, >99% wt.), poly(3,4-ethylenedioxythiophene) polystyrene sulfonate dispersion in water (PEDOT:PSS, M124) were purchased from Osilla (Sheffield, UK) and used as received. Iodine crystals (p.a.) were purchased in POCH (currently Avantor Performance Materials, Gliwice, Poland) and used without further purification. Chlorobenzene was purchased from Avantor Performance Materials (Gliwice, Poland), and used as received.

### 2.2. Thin Films-Deposition and Thickness Measurements

The iodine doping was conducted in chlorobenzene solutions of P3HT or P3HT:PCBM (1:1 wt.) blend of 10 mg/mL concentration. The iodine was introduced in various contents (0, 1, 5 or 10 % mol.) towards P3HT and, subsequently, prepared solutions were spin-coated on the quartz or glass substrates, at 1500 rpm, which resulted in the formation of thin films.

Thicknesses of thin films and the roughness of their surfaces were measured by the atomic force microscopy (AFM) technique, using AFM Topo-Metrix Explorer microscopy, working in a contact mode in the air, in the constant force regime. All obtained thicknesses of thin films, together with their root mean squares (RMS) of surface’s roughness are gathered in Table S1 in the Supplementary Information part.

### 2.3. Measurements Techniques

#### 2.3.1. UV-Vis-NIR Optical Investigations

Optical measurements were carried out using a two-beam UV-Vis-NIR, JASCO V-570 spectrophotometer, working with the Spectra Manager Program. Transmission (*T*%) and reflectivity (*R*%) spectra of thin films on quartz substrates were registered at room temperature, within the spectral range of 200–2500 nm. During the reflectivity measurements, a special two-beam reflectance arrangement was used, with an Al mirror in the reference beam, as a reflectance standard. Due to the small level of films reflectivity (5–8%) within the whole spectral range, the absorption coefficient (*α*) was calculated neglecting the reflectivity, using the simple equation [26]:

(1) $$\alpha =\left(\frac{1}{d}\right)\ln\left(\frac{1}{T\%}\right)$$

where *d* is films’ thickness

Moreover, the temperature (*T*) dependence of absorption coefficient, i.e., α(*T*) was obtained on the basis of transmission measurements at higher temperatures. All investigated thin films were subjected to a stepwise annealing in a special auto-controlled equipment of the JASCO spectrophotometer, which enabled the registration of transmission spectra, at precisely defined temperatures (±0.5 °C). The special in situ computer program was used to control the heating protocol and the temperature of investigated samples. Transmission spectra of thin films were measured within the range of temperature from 20 °C up to 210 °C, every 20 °C. Between steps, the temperature was gradually increased, with a rate of 2 °C/min; the short isothermal phase was used to stabilize the target temperature. After the last step, during annealing (the measurement at 210 °C), the samples were left in the spectrometer and then transmission spectra were registered at room temperature, once more.

#### 2.3.2. X-Ray Diffraction Studies

X-ray diffraction studies were performed using the D8 Advance diffractometer (Bruker, Karlsruhe, Germany) with Cu-Kα cathode (λ = 1.54 Å). The critical angle for conjugated polymers using copper radiation is ~0.17° (2) [27,28] and layer thickness of sample (~100 nm); for the 2D-GIWAXS setup, the 0.18° incidence angle was applied, which is just above the critical angle for polymer layer and below the critical angle for SiO_2_ support material. The scan rate was 1.2°/min with a scanning step of 0.02° in the range of 2.5° to 60° 2Θ (dwell time 1 s). Measurements were performed in 7 variations, using different φ (Phi) angle, which corresponded to the sample rotation. As a φ = 0°, a longer edge was set as parallel to the X-Ray beam direction. The esulting φ rotation (15, 30, 45, 60, 75 and 90°) was programmed with a resolution of 0.1° φ. Obtained 2D patterns (with width of 3° 2θ) for different φ angles were integrated to 1D patterns. Background subtraction, occurring from air scattering, was performed using DIFFRAC.EVA program.

### 2.4. Photovoltaic Cells—Preparation and Characterization

Devices with the bulk-heterojunction structure were prepared on ITO-coated glass substrates (6 pixels, each with area of 4.5 mm^2^). After cleaning the substrate with isopropanol in the ultrasonic bath, a thin film of PEDOT:PSS was deposited by spin coating. Solutions in chlorobenzene of the active layer were prepared by dissolving blends of each individual P3HT:PCBM (1:1 wt) with 5% or 10% mol. of iodine. Such prepared solutions were spin coated on the PEDOT:PSS layer, and, subsequently, an aluminum counter electrode was evaporated on the top of the blend thin film.

The impedance spectroscopy (IS) measurements were performed using the technique both in dark and illuminated conditions, with the precise RLC meter Agilent HP E4980A in the frequency range from 20 Hz to 1 MHz with the small signal voltage excitation of 20 mVrms. In order to identify the phenomenon of photo-generation of charges, experiments in the dark and under illumination (white cold LED COB with the electrical power of 10 W, viewing the angle 140°, color temperature 6500 K and luminosity 850 mL) were completed.

The *J-V* curves of obtained photovoltaic devices were measured by the PV Test Solutions Solar Simulator under the AM1.5 solar illumination and using the Keithley 2400 Source Meter SMU Instrument.

## 3. Results and Discussion

### 3.1. Optical Properties

#### 3.1.1. Iodine-Doped P3HT Thin Films

**Figure 1:**
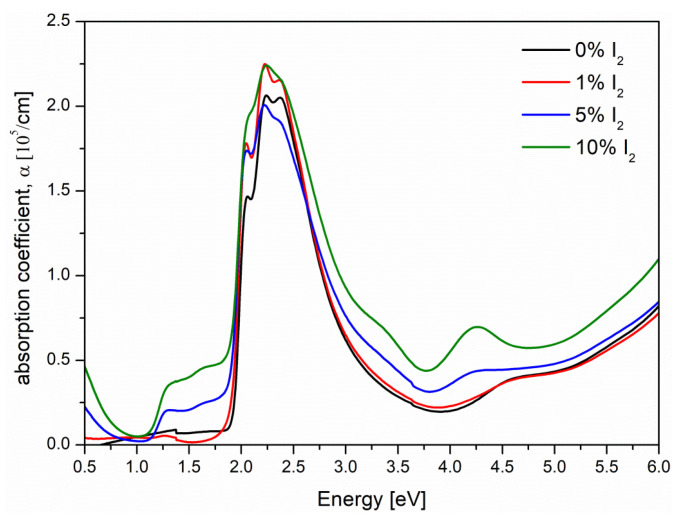
Absorption coefficient spectra, within the whole UV-Vis-NIR spectral range, of P3HT thin films with 0% (black), 1% (red), 5% (blue) and 10% (green) I_2_ mol. concentration.

Absorption coefficient (*α*) spectra, obtained at the room temperature, according to the Equation (1), for the neat and iodine (I_2_) doped P3HT films are presented in Figure 1.

The characteristic changes of absorption spectra of P3HT film at various iodine doping level (see Figure 1) were observed: (i) at about 1.5 eV, where the absorption was connected with the polaron states, (ii) within the range 2–3 eV where the strong peak was characteristic for *π*→π* electronic transitions and (iii) the absorption at about 4.2 eV, connected with the J-type aggregation (red shift of absorption peak) and intra-chain interactions [29]. The vibronic progression was clearly seen at the π→π* absorption band but changed with the iodine doping level. Some of these vibronic bands were very distinct, particularly for the neat P3HT film; however, in the case of the film with content of 10% iodine, these features were not evident. Thus, to find precisely the position of individual peaks, the second derivative method was used (i.e., minimum of the second derivative of absorption corresponds to the absorption maximum). Then, the vibronic progression of bands was deconvoluted, with the modified Fourier self-deconvolution and finite response operator (FIRO) methods [30]. Positions of all vibronic peaks: *λ*_A_^0-2^, *λ*_A_^0-1^, *λ*_A_^0-0^ in [nm] and [eV] and their intensities: *I*_A_^0-2^, *I*_A_^0-1^, *I*_A_^0-0^, obtained for all the spectra from Figure 1 are gathered in the Supplementary Information part in Table S2. Then, the exciton bandwidth (*W*) parameter was estimated (assuming a Huang-Rhys factor of unity) from the ratio of (0-0) and (0-1) absorbance peaks’ intensities, according to the formula [31,32,33]:

(2) $$\frac{I_{A}^{0-0}}{I_{A}^{0-1}}\approx {\left(\frac{1-024W/E_{p}}{1+0.73W/E_{p}}\right)}^{2}$$

using the $I_{A}^{0-0}$, $I_{A}^{0-1}$ values from Table S2 and where the phonon energy *E_p_* was involved with the main oscillator coupled to the electronic transition (a symmetric ring-stretching mode with energy 0.18 eV) [33]. The exciton bandwidth was connected with: the intra- and intermolecular excitonic coupling, electron-vibrational coupling and correlated energetic disorder, which led to the aggregate behavior in polymeric semiconductors. As shown in [31], the polymer P3HT can behave as both an H-type aggregate and a J-type aggregate, depending on the morphology (preparation method).

The edge of absorption, being the low-energy wing of the first low-energy band (the *π*→π* transition band of investigated thin films) was subjected to a more detailed analysis, which is the designation of absorption edge parameters, i.e., the energy gap width (*E*_G_) and the Urbach energy (*E*_U_). Overall, the value of energy gap of conjugated polymers depended on the length of conjugation in the polymer chain, while the Urbach energy was connected with the localized defect states within the energy gap. The absorption edges of investigated thin films exhibited an exponential region, which could be described by the Urbach relation [34]:

(3) $$\alpha \propto \exp\left(\frac{E}{E_{U}}\right)$$

**Figure 2:**
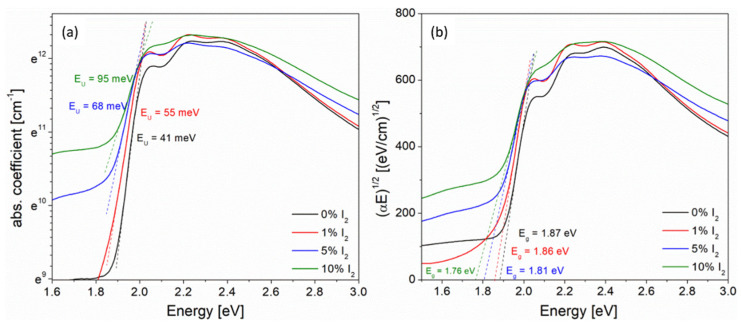
Absorption edges of iodine-doped P3HT thin films, used to obtain (**a**) the Urbach energy (**b**) the energy gap.

So, the *E*_U_ values of thin films were obtained based on the slope of the exponential edge, as it is seen in Figure 2a. The Urbach energy, as a “width of the band tail” occurring due to localized states within the energy gap, is caused by possible structural defects, such as a break, torsion or aberration of the polymer chains or molecules [35]; hence the “Urbach–like” behavior of absorption edges of investigated films was observed.

The values of energy gaps of neat and iodine-doped P3HT films were obtained based on the linear approximation to the energy axis, of the following relation [36]:

(4) $$\alpha \propto {\left(E-E_{G}\right)}^{2},$$

true for the energy *E > E*_G_.

This dependence, known as the Tauc relation, is typical for amorphous semiconductors, and is often used for polymers thin films and freestanding foils [37,38,39]. Since the X-ray diffraction studies for all investigated films before and after annealing demonstrated that the crystallinity was below 50% and that absorption edges were well fitted (as seen in Figure 2a), this dependence was used. The methods used to determine *E*_U_ and *E*_G_ is depicted in Figure 2a,b, respectively

**Figure 3:**
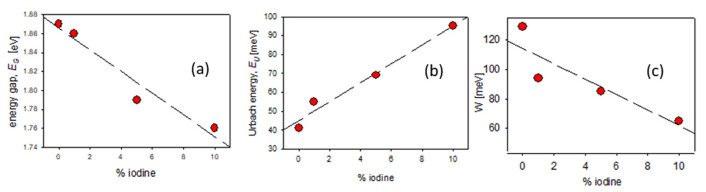
Optical parameters of neat and iodine-doped P3HT films (**a**) energy gap (**b**) the Urbach energy (**c**) the exciton bandwidth, as a function of % mol. content of iodine.

All obtained absorption edge parameters (*E*_U_, *E*_G_) and exciton bandwidths’ (*W*) values are gathered in Table S3 in the Supplementary Information part and are presented, as a function of the iodine % content, in Figure 3.

As it is seen in Figure 3a, the value of the energy gap decreased with the content of iodine, which could confirm the better conjugation after doping; simultaneously, the amount of defects increased, seen as an increase in the Urbach energy (Figure 3b), due to the localized defect states within the energy gap. Both the exciton bandwidth and energy gap decreased with an increase in iodine content (Figure 3c) which suggests the extension of the *π*-conjugation area in polymer chains.

Thermo-optical properties for the neat P3HT film were presented in [25], where this film was exposed to a stepwise heating and cooling and the changes of absorption edge parameters were discussed; the heat-inducted movement of elastic hexyl side chains and formation of defects at higher temperatures increased the free volume and decreased the order between the polymer chains [6]. Moreover, these changes turned out to be reversible and recurrent during annealing/cooling runs, while the energy gap was almost constant, which means that annealing up to 210 °C did not influence the conjugation in the main, rigid chain of P3HT film [23]. The same behavior during annealing was also observed in [38] for polymers with flexible octyloxy side chains.

**Figure 4:**
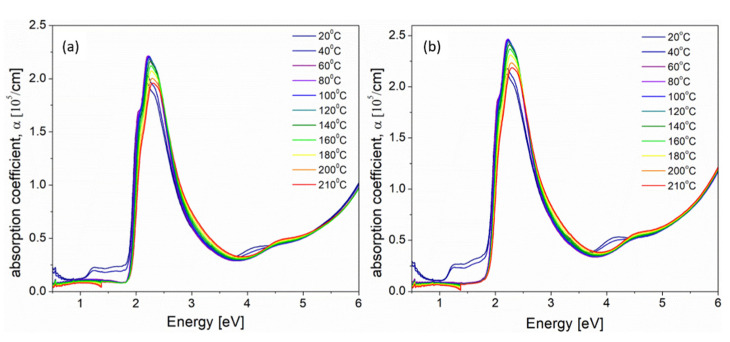
Absorption coefficient spectra, measured at different temperatures, within the whole UV-Vis-NIR spectral range, of iodine-doped P3HT thin films with (**a**) 5% and (**b**) 10% mol. concentration.

In this work, thermo-optical properties of iodine-doped P3HT films were investigated and transmission spectra of P3HT thin films with 5% and 10% of iodine (I_2_) mol. concentration were recorded in situ, every 20 °C, in the temperature range 20–210 °C. The absorption coefficient spectra, obtained for each temperature, are presented in Figure 4. These two concentrations of iodine were chosen for further experiments due to the best effect of power efficiency of such doped solar cells, as reported in [20].

**Figure 5:**
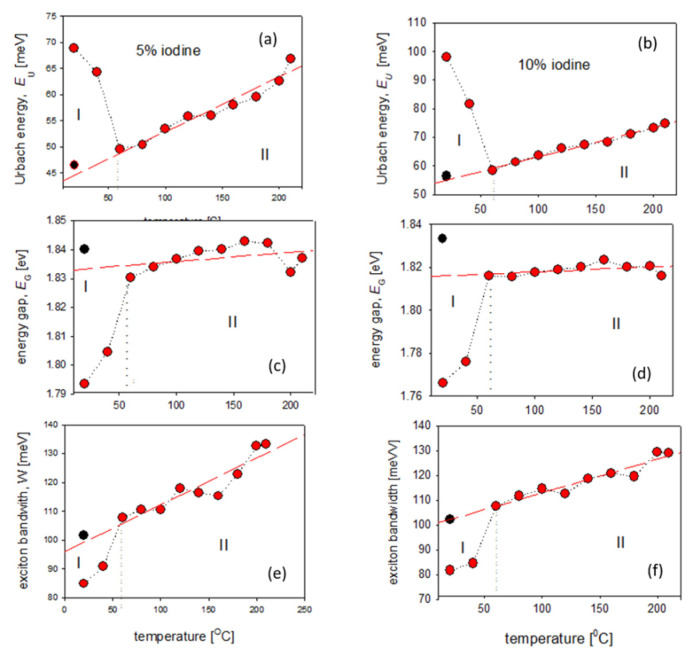
Temperature dependences of absorption edge parameters (*E*_U_, *E*_G_) and the exciton bandwidth (*W*) for 5% (**a**,**c**,**e**) and 10% (**b**,**d**,**f**) iodine-doped P3HT films; (black points in figures denote the values at 20 °C, after thermal treatment).

Then, using the Equations (3) and (4) and the same procedure, as it is seen in Figure 2, the Urbach energy and energy gap for each temperature were obtained. The method of determining absorption edge parameters, at representative temperatures, is shown in the Supplementary Information part, in Figure S1, together with all obtained optical parameters, gathered in Table S4. The temperature dependence of all obtained absorption edge parameters (*E*_U_, *E*_G_) and the exciton bandwidth (*W*) for 5% and 10% mol. concentration of iodine-doped P3HT films are presented in Figure 5. As it is seen in this figure, the content of iodine did not influence these temperature dependences of any calculated optical parameters.

The most characteristic changes of absorption coefficient spectra (Figure S1) and optical parameters (Figure 5) connected with the iodine escape process were seen at the temperature of 60 °C. The analysis of dependences of the absorption edge parameters (*E*_U_, *E*_G_) and the exciton bandwidth (*W*) on temperature, allowed to divide these runs into two stages:

(I) In the range 20–60 °C, the Urbach energy decreased and, simultaneously, the values of energy gap and exciton bandwidth increased. Annealing of iodine-doped P3HT films led to the releasing process of dopant atoms, connected with the disappearance of localized defect states within the energy gap (the lower Urbach energy) and the extinction of polaron bands and vibronic structure (see Figure 4), which results in the worse conjugation and higher values of energy gap and exciton bandwidth.
(II) Above 60 °C, during annealing up to 210 °C, the energy gap turned out to be almost constant, both for 5% (*E*_G_ ≅ 1.84 eV) and for 10% (*E*_G_ ≅ 1.82 eV) iodine-doped P3HT films, while the Urbach energy and exciton bandwidth slightly increased (Figure 5). Within this range of temperature, such behavior was similar to that for neat P3HT films [25], where the linear dependence of *E*_U_ on temperature was connected with the presence of flexible, hexyl side chains, while the almost constant value of *E*_G_ during heating confirmed unchanging conjugation of polymer main chains.

Then, after cooling to the room temperature, absorption spectra and optical parameters of investigated films were obtained once more. As it is seen in Figure 5 (black points) these values differed both from initials as from these parameters at 210 °C. Due to the relaxation of structural defects during the cooling process, the Urbach energies turned out to be smaller, while energy gaps and exciton bandwidths were larger than the values obtained at 60 °C, (when P3HT films were already without iodine atoms).

#### 3.1.2. Iodine-Doped P3HT:PCBM Blends Thin Films

**Figure 6:**
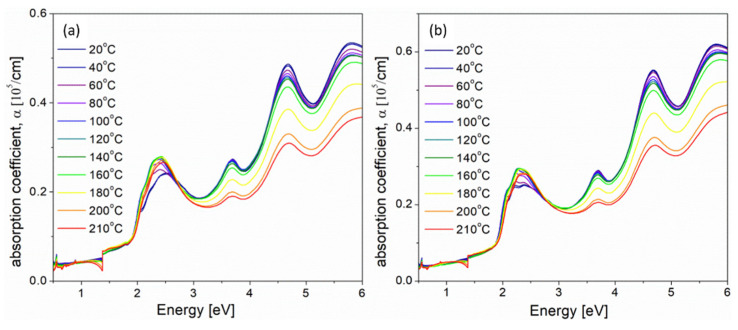
Absorption coefficient spectra, measured at different temperatures, within the whole UV-Vis-NIR spectral range, of iodine-doped P3HT:PCBM (1:1) blend thin films with (**a**) 5% and (**b**) 10% mol. concentration.

Absorption coefficient spectra obtained during annealing process of 5% and 10% iodine-doped blend thin films are presented in Figure 6a,b, respectively.

**Figure 7:**
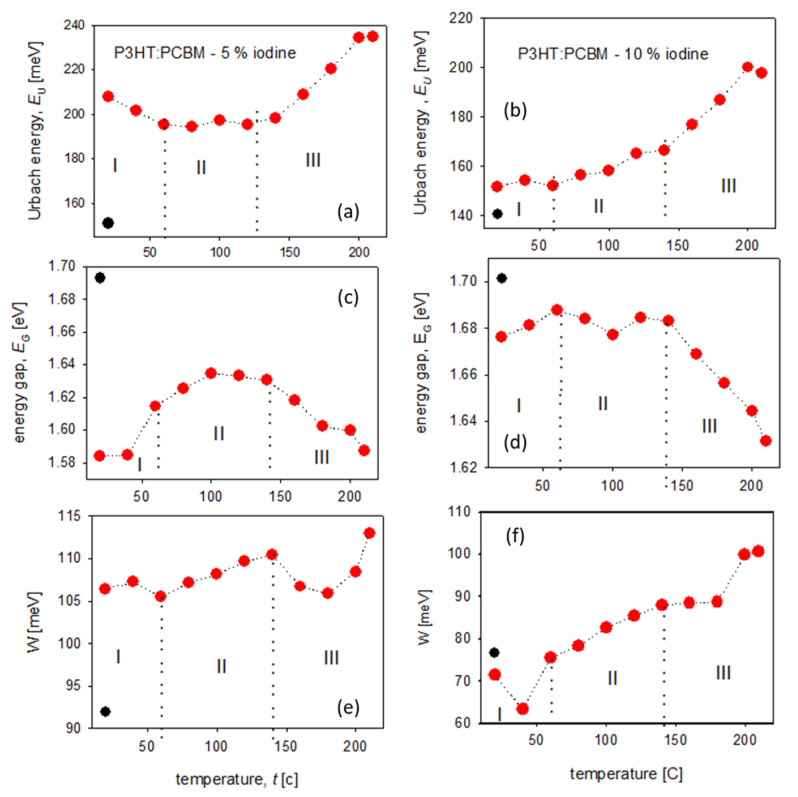
Temperature dependences of absorption edge parameters (*E*_U_, *E*_G_) and the exciton bandwidth (*W*) for 5% (**a**,**c**,**e**) and 10% (**b**,**d**,**f**) iodine-doped P3HT:PCBM blend films. (black points in figures mean the values at 20 °C, after thermal treatment).

Changes of absorption coefficient spectra (seen in Figure 6) under the influence of higher temperatures were clearly seen for all absorption bands. The band at about 2.5 eV was connected with the electron transitions between π→π* molecular orbitals of P3HT polymer, while three subsequent bands, seen in Figure 6, positioned at 3.70, 4.69 and 5.80 eV originated from the electron transitions in PCBM fullerene, as is described in [40]. The intensity of all bands connected with PCBM decreased during annealing, but their positions were unchanged. Decrease in the PCBM absorption bands during annealing can be explained by the formation of PCBM clusters in the P3HT matrix [23], while the bathochromic shift and increase in the P3HT band intensity with increasing temperature (seen in Figure 6 and Figure S2) are caused by an increase in P3HT crystallinity and the orderly stacking of polymer chains, respectively [23]. More information about the doped polymer:fullerene blend films behavior upon annealing may be obtained by analyzing the changes of absorption edge parameters and exciton bandwidth in higher temperatures. Similarly as for P3HT films, the way of determining absorption edge parameters, at representative temperatures, is shown in the Supplementary Information part, as Figure S2, together with all obtained optical parameters, gathered in Table S5. The temperature dependences of all obtained absorption edge parameters (*E*_U_, *E*_G_) and the exciton bandwidth (*W*) for 5% and 10% iodine-doped P3HT films are presented in Figure 7.

Contrary to the annealing process of iodine-doped P3HT films, where two main stages were determined (see Figure 5) the behavior of P3HT:PCBM blend film during thermal treatment was more complicated and connected not only with the release of iodine (Figure 7). Moreover, we could observe unexpected differences between these runs for 5% and 10% iodine-doped blends films. Generally, three stages during annealing process were obtained:

(I) The first region, from 20 to 60 °C, was connected with the gradual iodine escape process and, simultaneously, with the increase in P3HT order. For 5% iodine, the blend predominated the polymer ordering (decrease in the Urbach energy) while, for 10% content of iodine, these two processes were seen to be in equilibrium (almost constant *E*_U_). Since the polymer ordering is connected with an increase in P3HT crystallinity, such differences might be explained by the higher crystallinity of 10% mol. doped thin film at the beginning of annealing.
(II) The stage within the temperature range of 60–140 °C was related to the thermally- induced movements of flexible side chains of P3HT, while the conjugation in the main polymer chain was almost unchanged because the values of energy gaps within this stage were approximately on the same level. We could observe the slight increase in the Urbach energy and exciton bandwidth, together with the energy gap of about 1.63 eV for 5% and 1.68 eV for 10% iodine-doped blends films.
(III) Above 140 °C, the rapid increase in the Urbach energy and decrease in the energy gap was due to the phase separation process (which probably could have started above the PCBM glass transition temperature *T*_g_ = 124 °C [41]) and the formation of PCBM clusters, which have introduced the permanent defects.

Due to the degradation process of P3HT:PCBM blends under the influence of such high temperatures, all parameters obtained after cooling to the room temperature were difficult to interpret (see black points in Figure 7). This behavior is different from that of such thermal treatment undoped blend films [25], wich confirmed that the presence of iodine may introduce permanent structural changes.

### 3.2. X-Ray Diffraction Investigations

To closely investigate the morphology of active blends, X-ray diffraction measurements were performed for both neat and doped P3HT and P3HT:PCBM thin films. For the structural analysis, the unit cell parameter *a* is related to the short oligomer axis and *c* corresponds to the long axis of molecule, while *b* is related to the *π*-stacking period [28]. The calculated *d*-spacing for all thin films are gathered in Table S6.

#### 3.2.1. Iodine-Doped P3HT Thin Films

**Figure 8:**
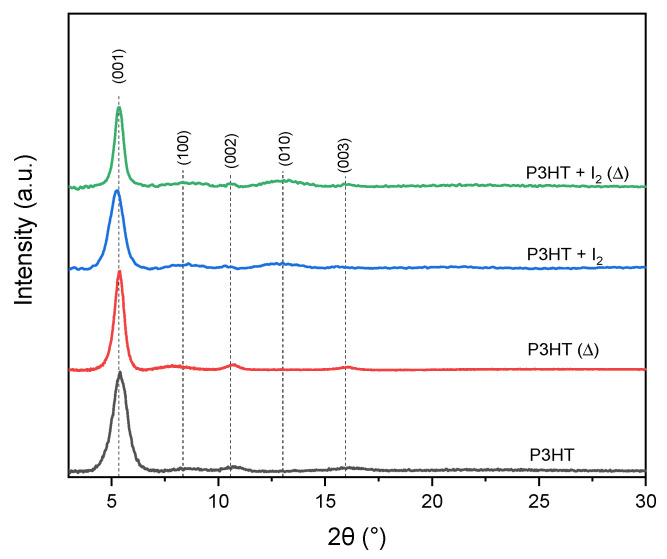
XRD patterns of neat P3HT (P3HT), 10% iodine-doped P3HT (P3HT + I_2_) before and after (Δ) thermal treatment (in 210 °C).

Registered diffractograms of neat and iodine-doped thin films before and after thermal treatment are presented in Figure 8.

For neat P3HT, the crystallinity of thin film was ~34% and slightly increased, after thermal treatment, to ~39%. Doped P3HT (10% mol. I_2_) thin films revealed higher crystallinity, 43%, which increased after thermal treatment to ~46%. Observed peak for the P3HT sample did not allow us to determine lattice parameters, due to lack of a (010) peak. However, a comparison of *d*-spacing of neat and iodine-doped samples showed that an introduction of 10% I_2_ slightly changed P3HT orthorhombic lattice. The *c* parameter (001 Miller index, long oligomer axis) of non-treated doped P3HT increased its dimension by 3.9%, while the enlargement for thermally treated film was only 1.5%. Annealing also slightly enlarged the chain axis (by 0.4%). In case of the *a* parameter (100 Miller index, short oligomer axis), introduction of I_2_ provoked a slight reduction in the *a* axis (by approx. 1%), while thermal treatment provoked its enlargement (9.4%) in comparison to the non-treated P3HT.

Application of both, iodine and annealing, also provoked an enlargement of *a* parameter (3.1%). The *b* parameter (010 Miller index) was not visible in any neat P3HT thin film, in contrary to iodine-doped layers, suggesting random orientation of lamellas. The *b* parameter for I_2_-doped P3HT decreased after annealing from 6.95 Å to 6.66 Å. The value decreased after thermal treatment, suggesting that this treatment allows material to obtain the higher arrangement. However, *π*-stacking peaks, which should be present at ~25.8° 2θ [42], were not visible, suggesting random or near-random orientation of lamellas in all samples. The *π*-stacking analysis of conjugated polymer systems is usually particularly inaccessible, because only the first-order peak is measurable (010 peak) [43]. Moreover, *d*-spacing calculated for peaks in the P3HT samples was gathered in Table S6 in the Supplementary Information part.

#### 3.2.2. Iodine-Doped P3HT:PCBM Blends Thin Films

**Figure 9:**
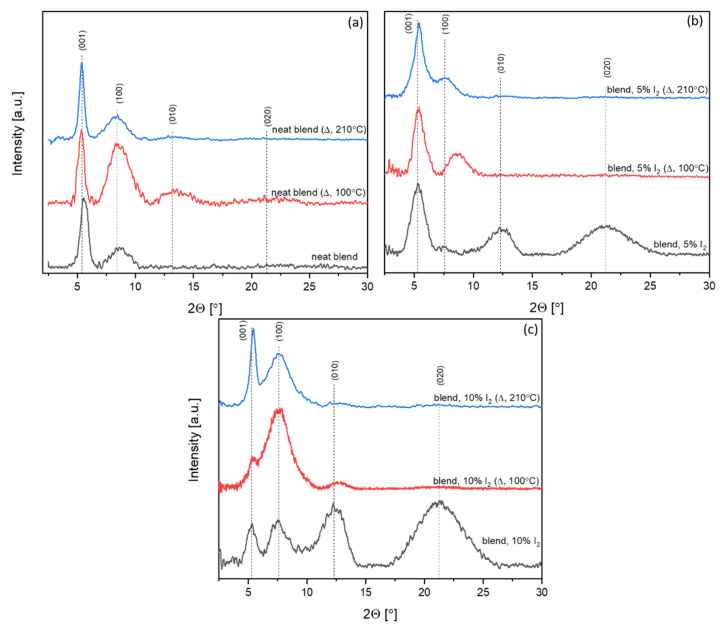
XRD patterns of (**a**) neat, (**b**) 5%, (**c**) 10% I_2_ doped P3HT:PCBM blend before and after thermal treatment.

Introduction of PCBM into P3HT resulted in the presence of only primary peaks of orthorhombic lattice (001 and 100), with much lower lattice parameters than in the case of neat P3HT (Figure 9).

Registered diffractograms revealed the high contraction of chain (long) axis (3.9%), while, for the short molecule axis, it was much lower (1%). For P3HT:PCBM, the enlargement of unit cells occurred, even in comparison to the neat P3HT after thermal treatment. Compared to the non-annealed neat blend, in the sample annealed at 210 °C, the chain axis enlarged by about 5.6%, while the short oligomer axis enlarged by 6.1%. Moreover, the presence of the (010) peak was detected, which suggests that introduction of PCBM combined with thermal treatment allowed us to obtain a higher order of the P3HT structure, with random orientation of lamellas (due to absence of π-stacking peak). In the neat active blend, annealed at 100 °C, enlargement of the P3HT lattice occurred in comparison to the non-treated blend. Moreover, the higher order of the P3HT structure was detected, due to the presence of (010) and (020) peaks. Intensity of the (010) peak was higher for the blend annealed at 100 °C than for the one at 210 °C, which may suggest disordering of the P3HT structure during long-term thermal exposition, which is consistent with the optical results presented above, where a degradation of blend was observed.

In non-annealed doped P3HT:PCBM blends (5% and 10% mol. I_2_ content), a high and broad (020) peak was visible, which corresponded to π-stacking. Peaks that corresponded to the *a* and *c* axis were smaller than peaks that corresponded to the *b* axis, while, in the sample, after thermal treatment, enlargement of (001) and (100) peaks occurred, with a simultaneous decrease in the intensity of (010) and (020) peaks. That might suggest the positive effect of iodine introduction on crystallization of P3HT. Crystallinity was higher for the neat blend thin film treated with 100 °C than for non-treated or treated with 210 °C and was 48%, 36% and 46%, respectively (see Table 1).

In all doped P3HT:PCBM samples, the crystallinity increased along with the iodine content, for each investigated heat treatment variant. An increase in the blend order was observed after annealing at 100 °C, which slightly decreased after treatment with 210 °C. This is consistent with the results, presented above, from in situ UV-Vis measurements, where ordering of P3HT and further blend degradation were observed, respectively.

### 3.3. Photovoltaic Response of BHJ Devices

#### 3.3.1. Photo-Active Impedance Spectroscopy (IS) Investigations

IS experiments of reference devices (ITO/PEDOT:PSS/P3HT:PCBM/Al) and organic solar cells with modified active layer (by adding iodine into the P3HT:PCBM solution) were conducted to define the influence of incorporated iodine on the photovoltaic phenomena and electrical parameters. Obtained spectra from IS measurements allowed for fitting the experimental data by an electrical equivalent circuit (EEC) and also on the estimate resistances and relaxation times (see Table S7) and capacitance behavior of organic solar cells. The proposed approach to the fitting of the obtained data from IS experiments was also mentioned in articles [44,45,46,47]; the same equivalent electrical circuit of the described phenomena that occurred in organic solar cells based on polymers from the polythiophene family was also used in [44,45,46,47].

**Figure 10:**
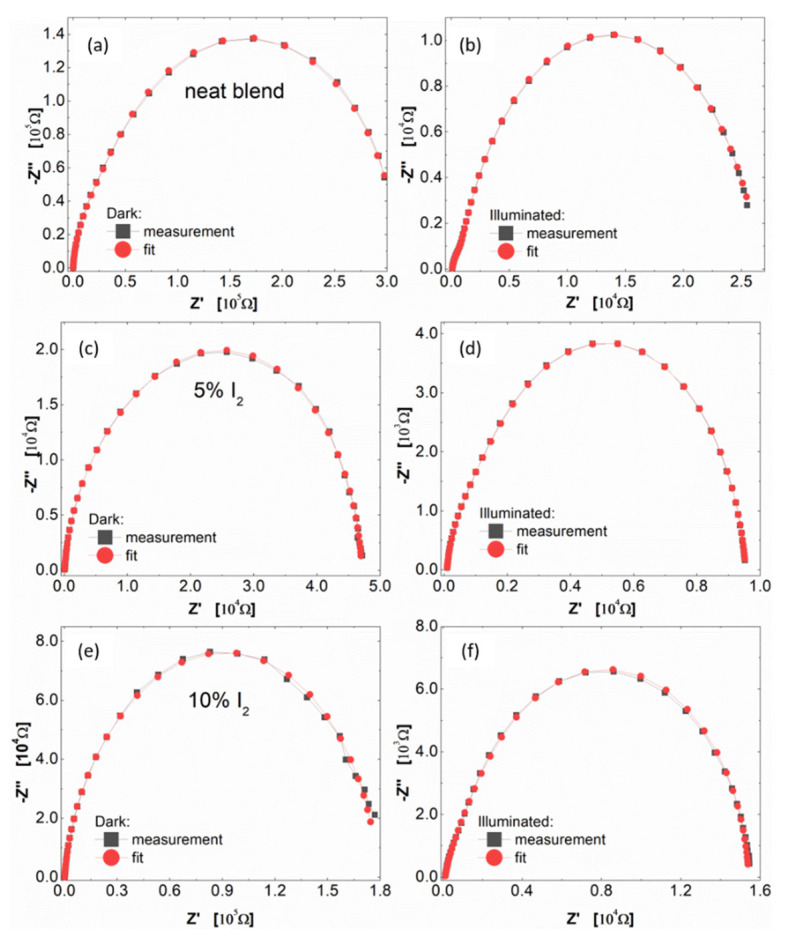
Electrochemical impedance spectra of standard organic solar cells (left side—in dark; right side -illuminated) with the active layer (**a**,**b**) undoped; (**c**,**d**) 5% (**e**,**f**) 10% iodine.

**Figure 11:**
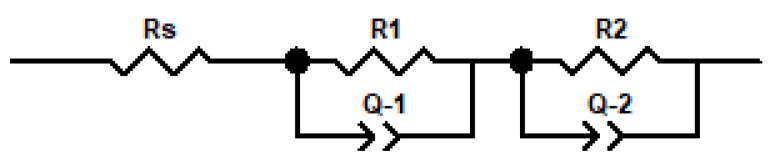
Electric equivalent circuit (EEC) used to model the photovoltaic devices impedance spectra in the dark and under illumination.

In Figure 10, the Nyquist plots of all working photovoltaic devises are presented. For all devices measured in dark conditions, one semicircle in the Nyquist plots (Figure 10a,c,e) was observed. On the other hand, for samples measured under illumination, two semicircles in the Nyquist chart (Figure 10b,d,f) could be easily detected. Furthermore, for all devices, the intensive reduction in the real part of impedance after irradiation in the relation to dark measurements was noticed. This is a confirmation of the intense photo-generation of charges in the active layer of devices. Obtained impedance spectra were analysed using electric equivalent circuit (EEC) modelling. The proposed structure of EEC was shown in Figure 11.

The reduction in all resistances after the illumination was clearly noticeable in the EEC modelling results, gathered in Table S7 in the Supplementary Information part. The most instant photo-generation effect, in the case of modified samples, was observed for the sample with 10% of iodine in active layer. Furthermore, a decrease in time constants (*τ*) in devices with modified active layer (with 5% and 10% iodine) vs. reference undoped sample, for measurements conducted under dark conditions, was observed (from 185.70 μs to 21.25 μs, and from 748.3 μs to 193.8 μs for *τ*_1_ and *τ*_2,_, respectively). Moreover, reduction in time constants (*τ*) for all devices after illumination was noticed. Such behaviour of the *τ* parameter confirms the fact of the improvement of photo-generation of chargers and charge transfer phenomena in modified devices, especially for the sample with 10% of iodine content.

#### 3.3.2. J-V Characteristics

- *Influence of iodine content*

**Figure 12:**
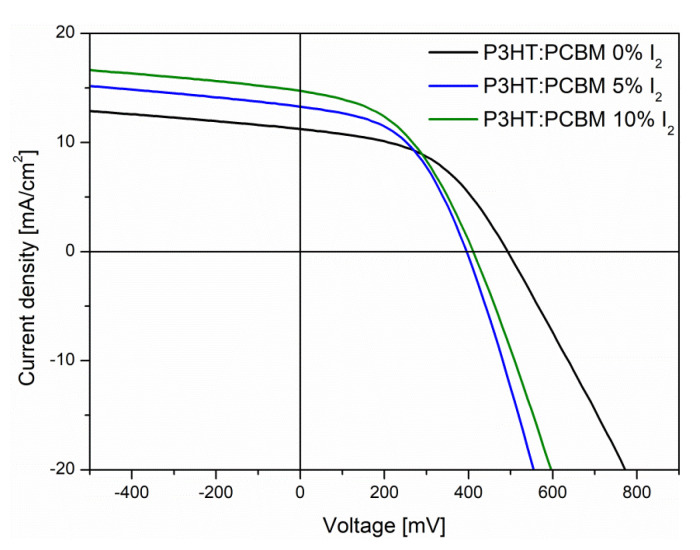
*J-V* characteristics of studied BHJ devices.

To correlate the results from optical, structural and IS measurements, presented above, the bulk-heterojunction (BHJ) photovoltaic cells were prepared and their current density–voltage *(J-V*) characteristics were registered. Firstly, the iodine content in the P3HT:PCBM blend films was considered. Since the most pronounced effects of iodine doping on optical spectra were visible when 5% and 10% mol. iodine was introduced, such active layers were used in BHJ devices (Figure 12) and compared with a neat reference. All active layers were annealed at temperature of 100 °C for 10 min.

Designated parameters (Table 2) showed an increase in device performance, along with an increase in iodine content. Such an enhancement was caused by a lower active layer series resistance (*R*_s_), which increased the short-circuit current density. This was most likely caused by a more favorable morphology of doped thin films, connected with their higher crystallinity, and is in agreement with IS results, where the improvement of photo-generation of chargers and charge transfer phenomena upon doping was observed. Introduction of 5% mol. of iodine caused a decrease in both series resistance (*R*_s_) and shunt resistance (*R*_sh_); however, the decrease in *R*_s_ was more pronounced; thus, an increase in power conversion efficiency from 2.24% to 2.41% was observed. Further, an increase in iodine content did not affect the series resistance; however, it increased the shunt resistance, lowering the current losses, and causing a further increase in PCE.

- *Influence of the thermal treatment*

**Figure 13:**
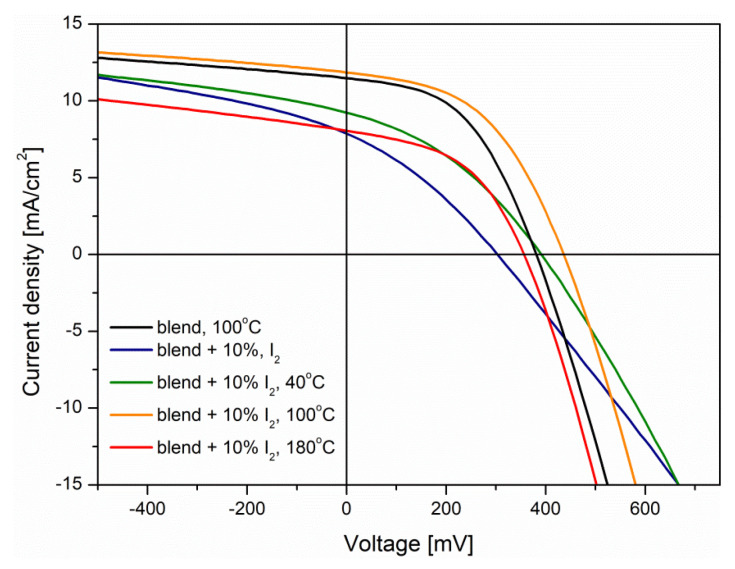
Current density-voltage characteristics of studied devices.

Subsequently, the thermal treatment effect of BHJ structure was considered. The 10% mol. iodine-doped active blends were tested in a non-treated form, and annealed at 40 °C, 100 °C and 180 °C. All registered current density–voltage characteristics (Figure 13) were compared with the results from the device with neat active blend (annealed at 100 °C).

Based on the registered *J-V* curves, all characteristic parameters of prepared photovoltaic cells were designated and gathered in Table 3.

Analysis of registered parameters revealed that the non-annealed, doped, P3HT:PCBM active blend provided much lower efficiency than a reference device with the neat active layer. This is associated with much smaller conductivity of such thin film (higher series resistance), resulting from low crystallinity of the conducting polymer (P3HT) [48]. Slight increase in the *J*_SC_ after annealing at 40 °C probably resulted from an increase in the polymer crystallinity, induced by heat-induced ordering of P3HT chains [4]. The device with doped active layer, annealed at 100 °C, showed further improvement of registered parameters, achieving higher efficiency (2.35%) than a reference (2.06%). Such enhancement was most likely caused by a lower donor–acceptor interface area, suppressing the non-radiative recombination voltage losses [49]. Apart from this, the orientation of P3HT chains took place, decreasing the series resistance, and increasing *R*_sh_. Further elevation of annealing temperature (180 °C), due to blend degradation, significantly lowered the device performance [41].

## 4. Summary and Conclusions

In conclusion, this work reported on the iodine (I_2_)-doped polymer (P3HT) and blended with the fullerene (PCBM) thin films and their thermo-optical and structural properties, towards photovoltaic applications in BHJ structures. Results of absorption and X-ray diffraction studies showed a positive effect of iodine dopant on the crystallinity of polymer and its blend, with fullerene, inducing a bathochromic shift of the low-energy absorption band and the decrease in energy gap. However, thermo-stability of these doped blend films can be a problem, particularly in the case of their photovoltaic applications, since the solar cells are exposed to the effect of higher temperatures.

Herein, presented results provide new information about the changes in optical and structural properties of doped polymer and polymer:fullerene blend films upon annealing. The novel method of in situ optical measurements of thin films, during their annealing, allows one to observe the changes of absorption spectra, connected with the iodine release and other thermal-inducted structural changes. Several processes that took place in the doped P3HT or its blend with PCBM thin films were distinguished. Temperature dependencies of the exciton bandwidth (*W*) and absorption edge parameters (*E*_U_, *E*_G_) were used to obtain:

- for the iodine-doped P3HT films, the temperature range of iodine escape (up to 60 °C). Above this temperature, the energy gap turned out to be almost constant, both for 5% (*E*_G_ ≅ 1.84 eV) and for 10% (*E*_G_ ≅ 1.82 eV) of iodine content, while the Urbach energy and exciton bandwidth slightly increased. These changes were connected with the presence of flexible side chains, while the almost constant value of *E*_G_ confirmed unchanging conjugation in polymer main chains.
- for iodine-doped blends films, three different stages: (i) up to 60 °C: the gradual iodine escape process and simultaneously the increase in P3HTcrystallinity; (ii) 60–140 °C: changes related to the thermally inducted movements of the flexible side chains of P3HT and the initiation of phase separation; (iii) above 140 °C: the gradual blend degradation, due to the formation of PCBM clusters, which introduced permanent defects.

These thermo-optical investigations confirmed that the presence of fullerene in blend with P3HT influenced the properties of polymer. Moreover, the content of iodine changed the structural order of blend films and their behavior during annealing.

The structural changes of neat and doped polymer and blend films were considered by X-ray diffraction studies, performed for samples annealed at 100 °C and 210 °C. In all doped P3HT:PCBM films, the crystallinity increased along with the iodine content, for each of the investigated heat treatment variants. An increase in the blend order was observed after annealing at 100 °C for all samples; the highest crystallinity (51%) was obtained for 10% iodine content blend film. Crystallinity decreased after treatment with 210 °C due to the blend thermal degradation processes.

Solar cells with these iodine-doped P3HT:PCBM active layers were investigated by the impedance spectroscopy (IS) and current density-voltage (J-V) characteristics. The highest power conversation efficiency (2.61 ± 0.13%) and the most effective photo-generation effect were detected for devices with the modified active layer by 10% of iodine. Thermal treatment of this photovoltaic device confirmed the fact that annealing at 100 °C is the best post-deposition strategy to improve solar cells parameters.

## Figures and Tables

**Table 1:** Crystallinity of investigated thin films after heat treatment.

Heat-Treatment	Neat P3HT:PCBM	P3HT:PCBM + 5% I_2_	P3HT:PCBM + 10% I_2_
non-annealed	36%	37%	39%
annealed at 100 °C	48%	49%	51%
annealed at 210 °C	46%	46%	49%

**Table 2:** Parameters of BHJ devices under illumination.

Layer	*V*_OC_ [mV]	*J*_SC_ [mA/cm^2^]	*FF*	PCE [%]	*R*_s_ [Ω]	*R*_sh_ [kΩ]
neat	465.2 ± 21.1	9.94 ± 0.91	0.48 ± 0.01	2.24 ± 0.29	354.89 ± 39.55	5.18 ± 0.75
5% I_2_	394.2 ± 4.4	12.95 ± 0.59	0.46 ± 0.02	2.41 ± 0.19	240.10 ± 33.59	3.87 ± 0.78
10% I_2_	405.1 ± 6.1	14.18 ± 0.59	0.44 ± 0.01	2.61 ± 0.13	246.69 ± 26.41	4.24 ± 0.88

*V*_OC_—open circuit voltage, *J*_SC_—short circuit current density*, FF*—fill factor, PCE—power. conversion efficiency, *R*_s_—series resistance, *R*_sh_—shunt resistance.

**Table 3:** Parameters of BHJ devices under illumination.

Layer (Blend)	*V*_OC_ [mV]	*J*_SC_ [mA/cm^2^]	*FF*	PCE [%]	*R*_s_ [Ω]	*R*_sh_ [kΩ]
net 100 °C	376.26 ± 3.7	11.14 ± 0.32	0.48 ± 0.01	2.06 ± 0.06	248.18 ± 6.59	6.49 ± 0.82
+10% I_2_	281.1 ± 12.3	7.68 ± 0.43	0.30 ± 0.01	0.66 ± 0.07	591.33 ± 24.91	2.62 ± 0.50
+10% I_2_-40 °C	380.0 ±8.0	8.80 ± 0.56	0.36 ± 0.01	1.24 ± 0.09	509.81 ± 26.60	2.61 ± 0.42
+10% I_2_-100 °C	430.7 ± 7.0	11.10 ± 0.53	0.48 ± 0.01	2.35 ± 0.13	271.03 ± 6.95	5.89 ± 0.25
+10% I_2_-180 °C	348.1 ± 9.2	7.83 ± 0.43	0.46 ± 0.01	1.28 ± 0.11	304.95 ± 15.76	3.97 ± 0.32

*V*_OC_—open circuit voltage, *J*_SC_—short circuit current density, *FF*—fill factor, PCE—power conversion efficiency, *R*_s_—series resistance, *R*_sh_—shunt resistance.

## Data Availability

The data presented in this study are available on request from the corresponding authors.

## Supplementary Materials

The following supporting information can be downloaded at: https://www.mdpi.com/article/10.3390/polym14050858/s1, Table S1. References [28,42,43,45,46,50,51,52,53] are cited in the Supplementary Materials. Thicknesses and *RMS* of surfaces’ roughness of polymer and blend films, Table S2. Positions and intensities of absorption bands of P3HT thin films neat and doped with various I_2_ mole ratios, Table S3. Optical parameters of iodine-doped P3HT films, Figure S1. Absorption coefficient spectra within the whole spectral range of P3HT iodine-doped films, Table S4. Temperature dependence of optical parameters of P3HT iodine-doped 5% and 10% films, Figure S2. Absorption coefficient spectra within the whole spectral range of the P3HT:PCBM blend iodine-doped films, Table S5. Temperature dependence of optical parameters of thin films of P3HT:PCBM blends iodine-doped 5% and 10% films, Table S6. *d*-spacing calculated for peaks in P3HT samples, Table S7. The results of modeling procedure (d-dark; i-illuminated).
