# Supplementary material for: Thermo-Optical and Structural Studies of Iodine-Doped Polymer: Fullerene Blend Films, Used in Photovoltaic Structures
Source: Polymers (Basel). 2022 Feb 22;14(5):858. doi: 10.3390/polym14050858 (PMC8912530; doi:10.3390/polym14050858)
Supplement: Supplementary file 1 [file polymers-14-00858-s001.zip › polymers-1563475-supplementary.pdf]

Supplementary Information

Thermo-optical and structural studies of iodine doped polymer:fulerene blend films. used in photovoltaic structures

Bożena Jarząbek[1*]. Paweł Nitschke[1*]. Marcin Godzierz[1]. Marcin Palewicz[2]. Tomasz Piasecki[2]. Teodor Paweł Gotszalk[2]

[1] Centre of Polymer and Carbon Materials. Polish Academy of Sciences. 34 M. Curie-Skłodowska Str.. 41-819 Zabrze. Poland

[2] Department of Nanometrology. Faculty of Electronics. Photonics and Microsystem. Wroclaw University of Science and Technology. 50-372 Wroclaw. Poland

[*] Correspondence : bozena.jarzabek@cmpw-pan.edu.pl; pnitschke@cmpw-pan.edu.pl

Table S1. Thicknesses and *RMS* of surfaces' roughness of polymer and blend films.

**Thin film**	***d*** [nm]	***RMS*** [nm]
neat P3HT	102	6
P3HT + 1% I_2	159	8
P3HT + 5% I_2	227	8
P3HT + 10% I_2	238	9
neat P3HT:PCBM		20
P3HT:PCBM - 100°C	98	10
P3HT:PCBM - 210°C		23
P3HT:PCBM +5% I_2		16
100°C	103	16
210°C		21
P3HT:PCBM + 10% I_2		16
100°C	106	11
210°C		38

As it is seen above. in **Table S-1**. the surfaces of all iodine doped P3HT films caused to be smooth (RMS within the range of 6-9 nm) while the thickness increased along with the iodine content (from 102 nm for the net P3HT film up to 238 nm for 10% of iodine mol. concentration). In the case of blends. the presence of iodine almost did not influence the thickness (from 98 nm for the net blend to 106 nm for blend with 10% of iodine content) moreover these values were constant during annealing. Thermal treatment of iodine doped blend films influenced their surfaces roughness. connected with the polymer crystallization and thermal inducted structural changes. The lowest values of RMS were obtained after annealing at 100 °C. while for these blend films after annealing up to 210 °C. the structural defects connected with phase separation caused the largest RMS of surface roughness. It is especially seen for blend film with 10% iodine. what means that the initial content of iodine influenced the blends' structure.

S-3.1.Optical investigations

Table S2. Positions and intensities of absorption bands of P3HT thin films neat and doped with various I_2 mole ratios

**I_2 mole** ratio [%]	**λ_A^{0-2}** [nm] / [eV]	**I_A^{0-2}** [a.u.]	**λ_A^{0-1}** [nm] / [eV]	**I_A^{0-1}** [a.u.]	**λ_A^{0-0}** [nm] / [eV]	**I_A^{0-0}** [a.u.]
0	524.0 / 2.41	0.89	554.0 / 2.21	0.90	602.0 / 2.03	0.64
1	526.0 / 2.36	1.49	558.0 / 2.22	1.55	606.0 / 2.05	1.23
5	*526.0* / 2.36*	0.80	558.0 / 2.22	0.86	606.0 / 2.05	0.72
10	*526.0* / 2.36*	0.93	554.0 / 2.21	0.97	*604.0* / 2.05*	0.80

*obtained using deconvolution

Table S3. Optical parameters of iodine doped P3HT films

**I_2 mole** ratio [%]	E_G [eV]	E_U [meV]	W [meV]
0	1.87	41	129
1	1.86	55	94
5	1.79	69	85
10	1.76	95	65

E_G - energy gap; E_U - Urbach energy; W – exciton bandwidth

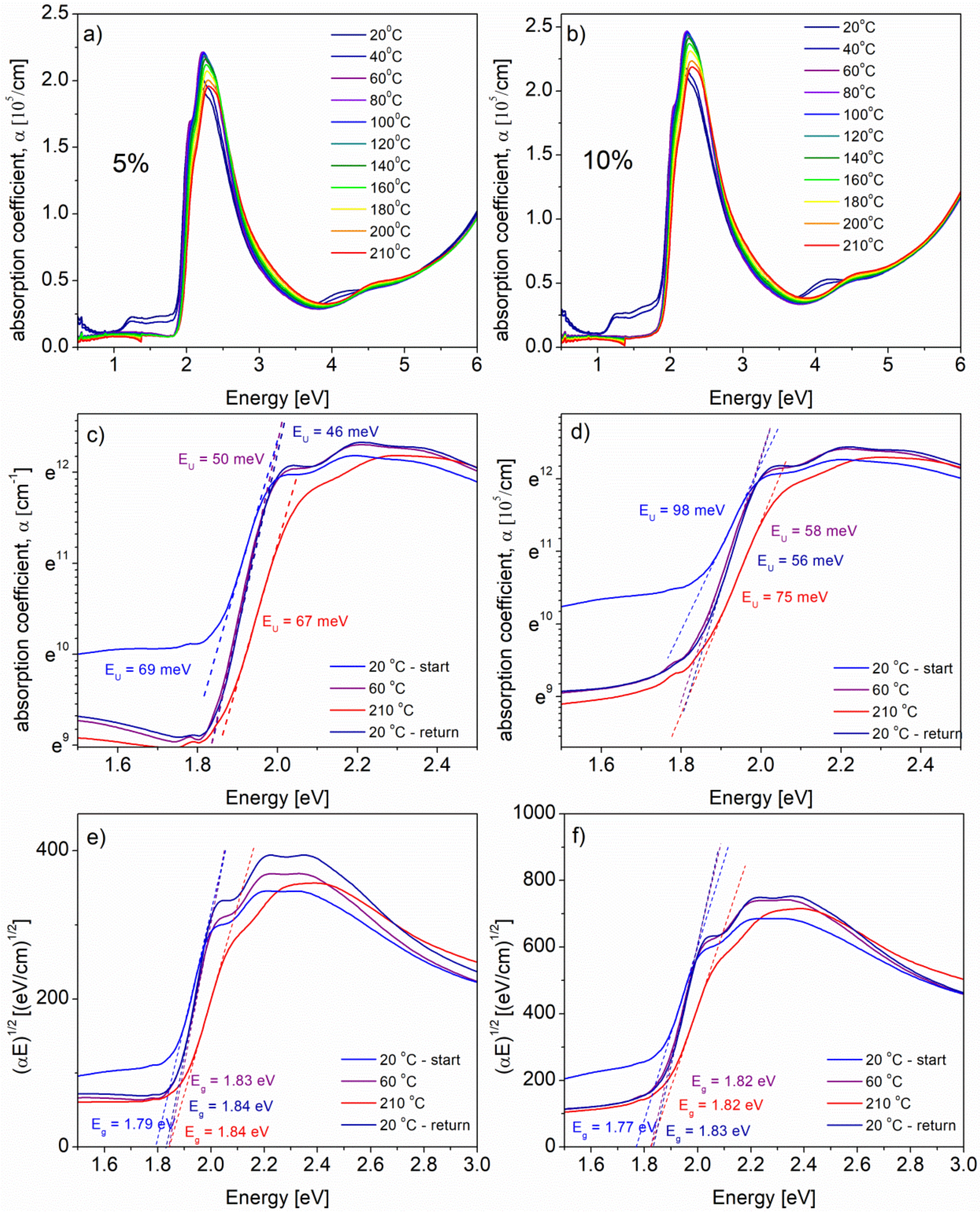

Figure S1. Absorption coefficient spectra within the whole spectral range of P3HT iodine doped films (a) 5% (b) 10% ; Absorption edge parameters (c. d) the Urbach energy; (e. f) the energy gaps. obtained of at chosen temperatures; (left side 5% and right side 10% iodine)

.

Table S4. Temperature dependence of optical parameters of P3HT iodine doped 5% and 10% films

**T** [°C]	**5%** mol. I_2			**10%** mol. I_2		
	E_U [meV]	E_G [eV]	W [meV]	E_U [meV]	E_G [eV]	W [meV]
20	69	1.79	85	98	1.77	82
40	64	1.80	91	82	1.78	85
60	50	1.83	108	58	1.82	108
80	50	1.83	110	61	1.82	112
100	53	1.84	110	64	1.82	115
120	56	1.84	118	66	1.82	113
140	56	1.84	116	67	1.82	119
160	58	1.84	115	68	1.82	121
180	60	1.84	123	71	1.82	120
200	63	1.83	133	73	1.82	129
210	67	1.84	133	75	1.82	129
20-return	46	1.84	102	56	1.83	102

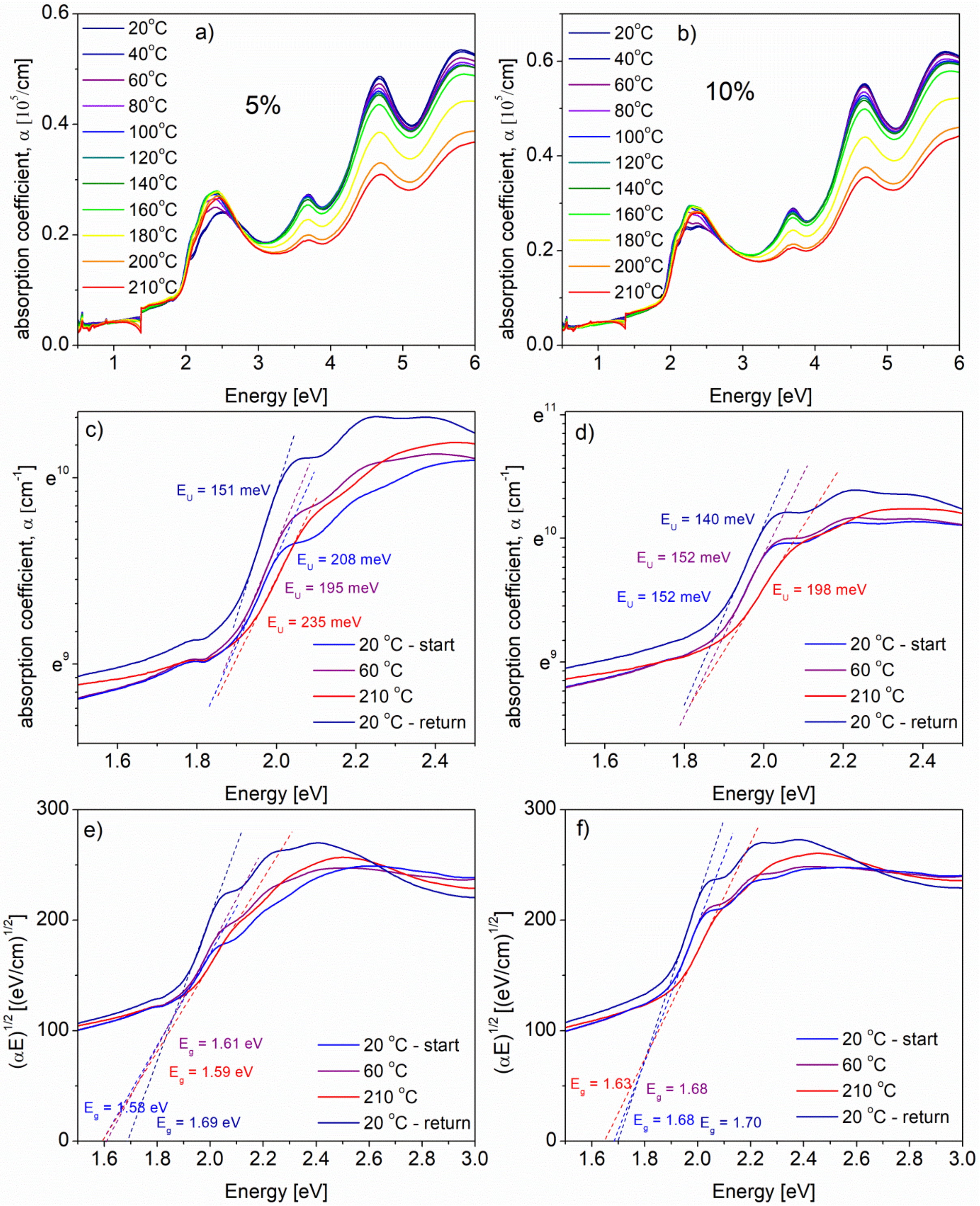

Figure S2. Absorption coefficient spectra within the whole spectral range of the P3HT:PCBM blend iodine doped films (a) 5% (b) 10% ; Absorption edge parameters (c. d) the Urbach energy; (e. f) the energy gaps. obtained of at chosen temperatures; (left side 5% and right side 10% iodine)

Table S5. Temperature dependence of optical parameters of thin films of P3HT:PCBM blends iodine doped 5% and 10% films

**T** [^{o}C]	**5%** mol. I_2			**10%** mol. I_2		
	E_U [meV]	E_G [eV]	W [meV]	E_u [meV]	E_G [eV]	W [meV]
20	208	1.58	106	152	1.68	71
40	201	1.58	107	154	1.68	63
60	195	1.61	105	152	1.69	75
80	194	1.63	107	156	1.68	78
100	197	1.63	108	158	1.68	83
120	195	1.63	110	165	1.68	85
140	198	1.63	110	166	1.68	88
160	209	1.62	107	177	1.67	88
180	221	1.60	106	187	1.66	89
200	234	1.60	108	200	1.64	100
210	235	1.59	113	198	1.63	101
20-return	151	1.69	92	140	1.70	77

S-3.2. X-ray diffraction investigations

From obtained WAXD patters. the peaks *d*-spacing and crystallite size were calculated using Bragg law (eq. 1) and Scherrer law (eq. 2).

$$d - spacing = \frac{n\lambda}{2\sin\theta} \quad (1)$$

where: n - positive integer (1); λ - X-Ray wavelength (1.5418 Å); Θ - peak position

$$Crystallite\ size = \frac{K\lambda}{FWHM \cdot \cos\theta} \quad (2)$$

where: K - Scherrer constant (0.89); λ - X-Ray wavelength (1.5418 Å); FWHM - the line broadening at half the maximum intensity after subtracting the instrumental line broadening. in radians; Θ - peak position.

For the structural analysis. the unit cell parameter a is related to the short oligomer axis and c corresponds to the long axis of molecule. while b is related to the π-stacking period [1–5].

Table S6. *d*-spacing calculated for peaks in P3HT samples

**Miller indices**	***d*-spacing. Å**			
	**(1) P3HT**	**(2) P3HT after thermal treatment**	**(3) non-treated P3HT + I_2**	**(4) P3HT + I_2 after thermal treatment**
**(001)**	16.30	16.37	16.93	16.55
**(100)**	10.31	11.28	10.21	10.63
**(002)**	8.27	8.21	8.57	8.45
**(010)**	-	-	6.95	6.66
**(003)**	5.42	5.52	5.69	5.58

We calculate crystallite size using the Scherrer equation (2) and the results are listed in **Table S-7**. As it can be seen, an increase of crystallite size occurs during neat P3HT and blends annealing. Similar phenomena was described by Privitera et al in [6]. Moreover, the introduction of iodine into P3HT film provokes strong crystallite growth.

Considering standard deviations, it should be noted that for blends annealed at 210°C, crystallites show only small growth compared to blends annealed at 100°C. It can be an effect of new, fine crystallite formation during annealing at 100°C, followed by its amorphization at 210°C. However, such conclusions require additional studies by means of temperature depended-XRD.

Table S7. Crystallite size determined for P3HT and its blends using the Scherrer equation

Material	Crystallite size at room temperature [nm]	Crystallite size after annealing at 100°C [nm]	Crystallite size after annealing at 210°C [nm]
**P3HT**	12 ± 3	-	16 ± 4
**P3HT + I_2**	29 ± 14	-	33 ± 17
**P3HT:PCBM**	10 ± 3	15 ± 11	16 ± 8
**P3HT:PCBM + I_2 (5%)**	7 ± 3	13 ± 8	15 ± 5
**P3HT:PCBM + I_2 (10%)**	4 ± 3	11 ± 7	14 ± 4

S.3-3. IS investigations

The structure of the electric equivalent circuit (EEC) was based on the research reported previously by Perrier et al [7] It consists of the series resistor Rs modelling the contacts and connections resistance and two parallel R_m-Q-m circuits allowing for convenient calculation of two time constants related to the electron-hole recombination lifetime and diffusion time of electrons [8] The impedance of the constant phase element in function of frequency is given by the formula:

$$Z_Q = \frac{1}{T(j\omega)^p}. \qquad \text{where } 0 \leq p \leq 1 \tag{3}$$

The constant phase element describes the imperfect frequency dependence of capacitance and is commonly used in modelling of electric properties of compound or unordered materials and devices. It is defined by two parameters T and p. For $p = 1$ CPE behaves like ideal capacitor. for $p = 0.5$ CPE describes electric properties of the diffusion process [9] Such an element (e.g.: Q-1. Q-2) is used for described situations when they are expected processes with imperfections at interfaces or distribution of relaxation times.

The time constant τ of the parallel R_m-Q-m (where m=1. 2) circuit may be calculated using following formula:

$$\tau = (RT)^{\frac{1}{p}} \tag{4}$$

where R is the resistance. T and p are the R-Q parameters. Two time constants were calculated from both R-Q circuits. The lower frequency one τ_2 corresponds to the effective lifetime related to the electron-hole recombination. where the high frequency one τ_1 represents the electrons diffusion time. The results of modelling procedure are presented in **TableS-8**

Table S8. The results of modeling procedure (**d**-dark; **i**-illuminated)

**I_2 mole** ratio [%]		**$\chi 2$ [× 10^{-6}]**	**Rs [Ω]**	**R1 [Ω]**	**Q-1-T [$s^p\Omega^{-1}$] [× 10^{-6}]**	**Q-1-P [–]**	**R2 [Ω]**	**Q-2-T [$s^p\Omega^{-1}$] [× 10^{-6}]**	**CPE2-P [–]**	**τ_1 [µs]**	**τ_2 [µs]**
**0%**	**d**	493	82.55	28323	0.0111	0.9394	2.83E+05	0.0068	0.9648	187.3	1543
	**i**	113	89.00	642	0.0484	0.8784	25890	0.0849	0.8525	7.390	761.9

**5%**	**d**	98	83.39	8441	0.0127	0.9341	38571	0.0086	0.9478	56.30	214.4
	**i**	140	93.93	910.2	0.0387	0.9094	8540	0.0298	0.9144	12.68	117.3
**10%**	**d**	406	98.86	9404	0.0219	0.9878	1.70E+05	0.0082	0.9131	185.7	748.3
	**i**	753	109.70	1438	0.1485	0.7855	13887	0.0213	0.9505	21.25	193.8

χ2- goodness of fit measure**; R**s - series resistance related to the contacts and connections; **R1- Q-1/ R2 – Q-2 -** parameters of resistance - constant phase element circuit related to τ_1 – diffusion time of electrons/τ_2 – electron-hole recombination lifetime. respectively. **Q-1-T/ Q-1-p** and **Q-2-T/ Q-2-p –** parameters of constant phase element 1 and 2.

References

1. Zajaczkowski, W.; Nanajunda, S.K.; Eichen, Y.; Pisula, W. Influence of alkyl substitution on the supramolecular organization of thiophene- and dioxine-based oligomers. RSC Adv. 2017, 7, 1664–1670. https://doi.org/10.1039/C6RA24740G.
2. Rivnay, J.; Mannsfeld, S.C.B.; Miller, C.E.; Salleo, A.; Toney, M.F. Quantitative Determination of Organic Semiconductor Microstructure from the Molecular to Device Scale. *Chem. Rev.* **2012**, *112*, 5488–5519. https://doi.org/10.1021/cr3001109.
3. Kronemeijer, A.J.; Gili, E.; Shahid, M.; Rivnay, J.; Salleo, A.; Heeney, M.; Sirringhaus, H. A selenophene-based low-bandgap donor-acceptor polymer leading to fast ambipolar logic. Adv. Mater. 2012, 24, 1558–1565. https://doi.org/10.1002/adma.201104522.
4. Xiao, X.; Wang, Z.; Hu, Z.; He, T. Single Crystals of Polythiophene with Different Molecular Conformations Obtained by Tetrahydrofuran Vapor Annealing and Controlling Solvent Evaporation. J. Phys. Chem. B 2010, 114, 7452–7460. https://doi.org/10.1021/jp911525d.
5. Tang, M.; Zhu, S.; Liu, Z.; Jiang, C.; Wu, Y.; Li, H.; Wang, B.; Wang, E.; Ma, J.; Wang, C. Tailoring π-Conjugated Systems: From π-π Stacking to High-Rate-Performance Organic Cathodes. Chem 2018, 4, 2600–2614. https://doi.org/10.1016/j.chempr.2018.08.014.
6. Privitera, A.; Righetto, M.; De Bastiani, M.; Carraro,F.; Rancan M.; Armelao, L.; Granozzi, G.; Bozio, R.; Franco, L. Hybrid Organic/Inorganic Perovskite−Polymer Nanocomposites: Toward the Enhancement of Structural and Electrical Properties. The Journal of Physical Chemistry Letters, 2017, 8, 5981-5986.
7. Perrier, G.; De Bettignies, R.; Berson, S.; Lemaître, N.; Guillerez, S. Impedance spectrometry of optimized standard and inverted P3HT-PCBM organic solar cells. Sol. Energy Mater. Sol. Cells 2012, 101, 210–216. https://doi.org/10.1016/j.solmat.2012.01.013.
8. Macdonald, J.R. *Impedance Spectroscopy*; Pergamon Press Ltd.: London, UK, 1992; Volume 20. ISBN 9780471831228.
9. Arredondo, B.; Romero, B.; Del Pozo, G.; Sessler, M.; Veit, C.; Würfel, U. Impedance spectroscopy analysis of small molecule solution processed organic solar cell. Sol. Energy Mater. Sol. Cells 2014, 128, 351–356. https://doi.org/10.1016/j.solmat.2014.05.050.
